# Changes in health-related quality of life and work-related outcomes for patients with mild-to-moderate ulcerative colitis receiving short-term and long-term treatment with multimatrix mesalamine: a prospective, open-label study

**DOI:** 10.1186/s41687-018-0046-5

**Published:** 2018-04-27

**Authors:** Mary Kaye Willian, Geert D’Haens, Aaron Yarlas, Ashish V. Joshi

**Affiliations:** 1 Shire, 725 Chesterbrook Blvd, Wayne, PA 19087 USA; 2 Inflammatory Bowel Disease Centre, Academic Medical Centre, University of Amsterdam the Netherlands, Meibergdreef, 91105 AZ Amsterdam, The Netherlands; 3 Optum, 1301 Atwood Avenue, Suite 311N, Johnston, RI 02919 USA

**Keywords:** Multimatrix mesalamine, Ulcerative colitis, Health-related quality of life, Work-related outcomes, Absenteeism, Presenteeism, Productivity, Inflammatory bowel disease

## Abstract

**Background:**

Ulcerative colitis (UC) is associated with lower health-related quality of life (HRQoL), and with disease activity predicting lower HRQoL and worse work-related outcomes. The current study examined the burden of UC on patients’ HRQoL, as well as changes in patients’ HRQoL and work-related outcomes following short-term and long-term treatment with multimatrix mesalamine, and their correspondence with changes in disease activity.

**Methods:**

Data were from an open-label, multinational, prospective trial (ClinicalTrials.gov identifier: NCT01124149) of 717 adults with active mild-to-moderate UC who were treated with 4.8 g/day multimatrix mesalamine tablets once daily for eight weeks (acute phase). Four-hundred sixty-one patients who achieved partial or complete clinical and endoscopic remission subsequently received treatment with daily 2.4 g/day multimatrix mesalamine for 12 months (maintenance phase). At baseline, Week 8, and Month 12, patients were administered patient-reported outcomes (PRO) measures of HRQoL (the SF-12v2® Health Survey [SF-12v2] and Short Inflammatory Bowel Disease Questionnaire) and work-related outcomes (Work Productivity and Activity Impairment questionnaire, UC-specific version). SF-12v2 scores were compared to the U.S. general population using Analysis of Variance models to assess burden of UC on HRQoL. Mixed-effects repeated-measures models compared PRO scores across visits to assess change in PRO scores over time. Correlations examined the correspondence of changes in PRO scores with changes on a modified UC disease activity index (UC-DAI).

**Results:**

Baseline burden of disease observed on all SF-12v2 domains was partially eliminated at Week 8 and completely eliminated at Month 12. Statistically significant improvements from baseline were observed at both Week 8 and Month 12 for all PRO scores (all *P* < 0.001). Decreases in UC-DAI scores significantly predicted improvements in PRO scores during the acute treatment phase.

**Conclusions:**

Patients with UC receiving daily multimatrix mesalamine treatment showed significant improvements in all measured domains of HRQoL and work-related outcomes. Patients who achieved partial or complete clinical and endoscopic remission maintained these improvements for most of these domains over 12 months with continued daily treatment. Changes in HRQoL and work-related outcomes were inversely related to changes in disease activity. Findings support the effectiveness of multimatrix mesalamine for improving, and sustaining improvements, in HRQoL and work-related outcomes.

## Background

Ulcerative colitis (UC), a type of inflammatory bowel disease (IBD), is characterized by chronic inflammation and ulceration of the colon. Symptoms commonly associated with active UC include a frequent need to defecate, the presence of blood in stools, abdominal pain or cramping, and fatigue. The presence, frequency, and severity of these and other symptoms are predictive of health-related quality of life (HRQoL). Numerous studies measuring the burden of UC on HRQoL relative to the general population have found deficits in all measured aspects of HRQoL [1–6]. Several studies have identified UC disease activity as having a strong inverse relation with HRQoL [1, 3, 4, 7–17], while clinical trials of treatment interventions for patients with UC have found increases in HRQoL in as soon as three weeks, with improvements being maintained for at least 12 months with continued treatment [2, 18–21].

UC also exerts a burden on patients’ work-related outcomes, including employment and disability status, frequency and length of illness-related absenteeism, and productivity. For example, patients with UC have rates of unemployment and work disability that are typically two- to three-times higher than matched general population controls [22–26], while increased UC disease activity predicts higher rates of work disability, increased absenteeism, and decreased work productivity [10, 11, 16, 27–29]. We found no studies of patients with UC that reported analyses of treatment effects on work-related outcomes from a validated multidimensional measure. The analysis presented here aims to close this gap by directly assessing changes in work-related outcomes for patients with UC receiving short-term and long-term treatment.

The current analysis examines the burden of UC on HRQoL, as well as the impact of short-term (acute) and long-term (maintenance) treatment on HRQoL and work-related outcomes for patients with active mild-to-moderate UC who received multimatrix mesalamine tablets in a prospective open-label trial [30]. At the start of the trial, patients with active mild-to-moderate UC received daily treatment with 4.8 g/day of multimatrix mesalamine for eight weeks. Patients who achieved either partial or complete clinical and endoscopic remission at the end of this phase received an additional 12 months of daily maintenance treatment with a lowered dose (2.4 g/day) of multimatrix mesalamine. Patients’ HRQoL and work-related outcomes were assessed at the start and the end of both treatment phases.

Based on previous studies measuring HRQoL of patients with UC receiving multimatrix mesalamine [2, 21], we expected to observe below-average levels of HRQoL at baseline, improvements in HRQoL (in concordance with improvements in disease activity) during the acute phase, and maintenance of these improvements with continued treatment through the 12-month maintenance phase. Additionally, we hypothesized that work-related outcomes, like HRQoL, would show improvement following acute treatment, and that these improvements would not deteriorate through the 12-month maintenance treatment period.

## Methods

### Study design and sample

**Fig. 1:**
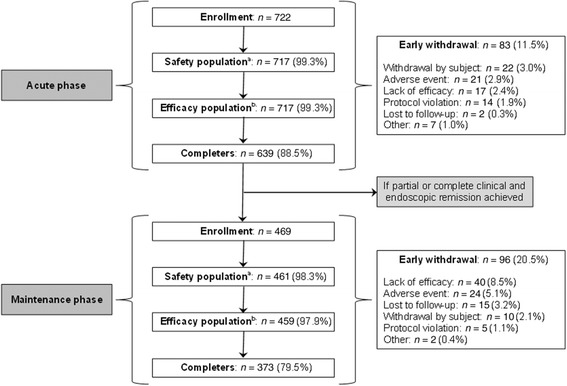
Flow chart for disposition of trial patients. ^a^The safety population within each phase was defined as all patients who took at least one dose of the investigational product during that phase. ^b^The efficacy population within each phase was defined as all patients who took at least one dose of the investigational product and had at least one post-dose efficacy assessment during that phase

Data for these post hoc analyses were from an open-label, multicenter, prospective trial of multimatrix mesalamine treatment for adult patients with active mild-to-moderate UC [31]. Patients from 14 countries participated in this trial, covering regions of North America (Canada, United States [US]), South America (Colombia), Europe (Belgium, Czech Republic, France, Hungary, Ireland, Poland, Romania, Spain, and United Kingdom), Africa (South Africa), and Asia (India). The trial consisted of two phases: an eight-week acute phase, during which patients were treated with 4.8 g/day of multimatrix mesalamine once daily (QD), followed by a 12-month maintenance phase, during which patients received 2.4 g/day of multimatrix mesalamine QD. The primary objective of this trial was to examine the degree to which patients’ clinical and endoscopic remission status at acute phase completion predicted their remission status at the end of the maintenance phase, the results of which have been published [30]. Assessing improvement in patients’ HRQoL and work-related outcomes were tertiary objectives of the trial. A CONSORT-style flowchart of the trial design, including patient disposition within each treatment phase, is presented in Fig. 1.

Enrollment in the trial was restricted to patients aged ≥ 18 years who were experiencing an acute flare or who had been newly diagnosed with active mild-to-moderate UC at screening, as defined by a total score of 4–10 (inclusive) on the modified Ulcerative Colitis Disease Activity Index (UC-DAI) [31] with an endoscopy score of ≥ 1 and a Physician’s Global Assessment (PGA) of ≤ 2 (i.e., not severe) at screening. Patients with a history of biologic use or who had used systemic or rectal steroids less than four weeks prior to screening were excluded from this trial. Of 892 patients screened, 722 enrolled in the trial. A total of 717 patients comprised the acute phase efficacy population, which was defined as patients who took at least one dose of multimatrix mesalamine and had at least one post-dose efficacy assessment during the acute phase (hereafter referred to as the “acute phase sample”). The data analyzed here were from patient-reported outcome (PRO) measures administered at three visits during the acute phase: post-screening baseline visit, Week 3 visit (for a UC-specific version of the Work Productivity and Activity Impairment questionnaire [WPAI:UC] only), and the Week 8 visit that occurred at Week 8 for patients who completed the acute phase or at the early withdrawal visit for patients who withdrew from the trial prior to Week 8.

Patients who at Week 8 were classified as being in either complete remission or partial remission (as defined using UC-DAI component and total scores) were eligible to enroll in the maintenance phase. From among 472 eligible patients at Week 8, a total of 469 enrolled in the maintenance phase. Of these, 459 patients were included in the maintenance phase efficacy population, defined in this trial as patients who took at least one dose of multimatrix mesalamine and had at least one post-dose efficacy assessment during the maintenance phase (hereafter referred to as the “maintenance phase sample”). The Week 8 visit of the acute phase was treated as the initial visit for the maintenance phase. PRO measures were also administered at the Month 12 visit, which occurred at Month 12 for patients who completed the maintenance phase or at the early withdrawal visit for patients who withdrew from the trial prior to Month 12.

This trial was approved by Institutional Review Boards at each site and included only patients who provided written informed consent at screening. The trial was assigned the ClinicalTrials.gov identifier NCT01124149.

### Measures

#### Disease activity measure

Clinical and endoscopic activity was assessed using a modified version of Sutherland’s UC-DAI.^1^ The UC-DAI total score was calculated as the sum of ratings on four components: stool frequency, rectal bleeding severity, mucosal appearance, and physician global assessment (PGA) of disease severity. Ratings for all components range from 0 to 3, and so total UC-DAI scores range from 0 to 12, with higher scores indicating greater disease severity.

Within this trial, complete remission was defined as a modified UC-DAI score ≤ 1 with scores of 0 points for both stool frequency and rectal bleeding severity and at least a one-point reduction in their mucosal appearance score from baseline. Partial remission was defined as a modified UC-DAI score ≤ 3 with the sum of stool frequency and rectal bleeding severity item scores ≤ 1, and not in complete remission.

#### PRO measures

Generic HRQoL was measured using the SF-12v2® Health Survey (SF-12v2), which is a 12-item, self-report survey of functional health and well-being with a four-week recall period [32]. The SF-12v2 captures eight domains of physical and mental HRQoL: Physical functioning (PF), Role limitations due to physical health problems (RP), Bodily pain (BP), General health perceptions (GH), Vitality (VT), Social functioning (SF), Role limitations due to emotional health problems (RE), and Mental health (MH). Weighted summations of domain scores are used to compute physical component summary (PCS) and mental component summary (MCS) scores, which provide a broader metric of physical and mental health, respectively. All SF-12v2 scores are norm-based, having been standardized based on scores from a national probability sample of 6012 non-institutionalized adults in the US who participated in a 2009 Internet-based survey conducted by QualityMetric, Inc. [32]. They are expressed as *T*-scores, with a mean of 50 and a standard deviation (SD) of 10. Higher values for all domains, as well as for PCS and MCS scores, reflect better HRQoL.

Disease-specific HRQoL was measured using the Short Inflammatory Bowel Disease Questionnaire (SIBDQ), which is a 10-item, self-report survey of the impact of respondents’ IBD (in this case, UC) on their HRQoL over the previous two weeks [33]. The SIBDQ has been shown to be reliable, responsive, and valid for assessing disease-specific HRQoL among UC patient populations [34, 35]. The SIBDQ yields scores on four domains – Bowel symptoms, Systemic symptoms, Emotional function, and Social function – and a total score that is calculated from responses to all items. Scores on the Bowel symptoms and Emotional function domains (three items each) range from 3 to 21 points, while scores on the Systemic symptoms and Social function domains (two items each) range from 2 to 14 points. Total score ranges from 10 to 70 points. For all domains and total score, higher values reflect better HRQoL.

Work-related outcomes were measured using the WPAI:UC, which is a six-item, self-report survey of the impact of a respondent’s UC on his/her work productivity and on non–work-related activities, such as shopping and child care, over the previous seven days [36]. Responses are used to calculate four domain scores: Absenteeism (work time missed due to a patient’s UC), Presenteeism (impairment while working due to a patient’s UC), Overall work impairment (overall productivity loss, accounting for both absenteeism and presenteeism, due to a patient’s UC), and Activity impairment (impairment in non-work activities due to a patient’s UC). Patients who report not being employed during the previous week receive scores only for the Activity impairment domain. All domain scores are expressed as percentages (ranging from 0 to 100%), with lower values indicating less impairment due to UC.

### Statistical analysis

The acute phase sample was included in analytic models of scores from acute phase visits only (i.e., baseline, Week 3, and Week 8). The maintenance phase sample was included in models of scores from maintenance phase visits (i.e., Week 8 and Month 12) and in models of scores from both acute and maintenance phase visits (i.e., Baseline, Week 3, Week 8, and Month 12).

The significance level of all statistical tests reported here were based on α = 0.05 (two-tailed). When simultaneously testing multiple pairwise comparisons of means within a model (e.g., testing pairwise differences in an outcome between each of three visits), family-wise error rate was controlled by adjusting *P*-values using Bonferroni’s method (i.e., dividing α by the number of tests conducted).

#### Baseline sample characteristics

Descriptive statistics for demographic and clinical characteristics and baseline scores on PRO measures were computed for patients in the acute phase and maintenance phase samples. Means and SDs were calculated for each continuous variable, while frequency and percentage were calculated for each categorical variable.

#### Burden analysis

To estimate the pre-treatment burden that UC placed on generic HRQoL, baseline SF-12v2 scores of patients in the acute phase sample were compared to SF-12v2 scores of a probabilistic US general population normative sample collected from QualityMetric, Inc.’s 2009 Internet-based survey [32]. To control for potential differences in age and gender distributions in the two samples, the US general population benchmark sample was matched to the age and gender of the acute phase sample using separate least squares multiple regression models for each SF-12v2 domain and both PCS and MCS scores. The matching of gender was conducted by applying the distribution of males to females from the trial sample directly to the benchmark sample, while age matching was based on applying the distribution of discrete age levels – 18 to 24, 25 to 34, 35 to 44, 45 to 54, 55 to 64, 65 to 74, and > 74 years – from the trial sample to the benchmark sample. Estimated mean scores were then derived for the US general population sample when using weights corresponding to these distributions of gender and age. Univariate analysis of variance (ANOVA) models for each SF-12v2 score, with sample as an independent variable, tested for significant differences between trial patients’ mean scores at baseline and estimated mean scores from the US population.

The degree to which the magnitude of burden changed following treatment in the acute phase and maintenance phase was examined by comparing SF-12v2 mean scores of the acute phase sample at Week 8 and SF-12v2 mean scores of the maintenance phase sample at Month 12, respectively, to scores from the age- and gender-matched US general population norm sample using the same statistical techniques described above for analysis of pre-treatment burden.

#### Impact of treatment on PRO measures

Analyses of SF-12v2, SIBDQ, and WPAI:UC mean scores across visits were conducted using separate mixed-effects models for repeated measures (MMRM) for each outcome. For each MMRM, subject was treated as random effect while visit was treated as a fixed, repeated measures effect. An unstructured covariance matrix was used for residuals. No models failed to converge using this approach, and examination of fit statistics (i.e., Akaike’s information criterion) showed a good model fit using this approach when compared to other common covariance matrices (homogenous and heterogeneous Toeplitz, First-Order Autoregressive, and compound symmetry).

SF-12v2 and SIBDQ domain and summary scores at baseline and Week 8 were analyzed using MMRM. Since this analysis compared means across two timepoints, Cohen’s *d*_*z*_ effect sizes for standardized mean differences in repeated measures, as well as 95% confident intervals (CIs) for effect size estimates, were calculated for mean changes from baseline to Week 8. Effect sizes were calculated using the following equation:

$$ {d}_z=\frac{Mean_1-{Mean}_2}{\sqrt{\left({s}_1^2+{s}_2^2\right)-\left(2\ast {r}_{12}\ast {s}_1\ast {s}_2\right)}}, $$

where *r*_12_ is the correlation between the outcome value at time 1 (Baseline) and time 2 (Week 8). The 95% CIs around each *d*_*z*_ were estimated using a bias-corrected-and-accelerated (BCa) bootstrap method [37], based on 10,000 random resamples. The magnitude of change for each comparison was interpreted following Cohen’s guidelines: *d* = 0.2: small effect; *d* = 0.5: medium-sized effect; and *d* = 0.8: large effect [38]. The importance of reporting effect sizes for interpreting differences in means has been highlighted by several researchers [39–41].

MMRM were also used to analyze WPAI:UC mean scores across baseline, Week 3, and Week 8 visits in acute phase sample patients; SF-12v2 and SIBDQ mean scores across baseline, Week 8, and Month 12 visits for maintenance phase sample patients; and WPAI:UC mean scores across baseline, Week 3, Week 8, and Month 12 visits for maintenance phase sample patients. For models with statistically significant differences among visits, post hoc tests using Bonferroni-adjusted *P*-values were conducted to examine pairwise comparisons of means between visits.

#### Remission sustainer analysis

Results from analyses previously conducted using these data [30] showed that approximately 30% of the maintenance phase sample were no longer in partial or complete remission at Month 12. This led to conjecture that a lack of sustained improvements from Week 8 to Month 12 may be observed for some PRO outcomes due to the presence of patients who did not sustain remission at Month 12. To test this hypothesis, we conducted a post hoc exploratory analysis examining whether changes in PRO outcomes from Week 8 to Month 12 differed between patients who sustained remission at Month 12 and patients who were not in remission at Month 12. Each PRO domain and summary score within the maintenance phase sample were analyzed using a MMRM model, with subject as a random effect and with maintenance phase visit (Week 8, Month 12), sustainer status at Month 12 (remission, not in remission), and the visit * sustainer status interaction as fixed effects. Evidence testing this hypothesis would be found by examining the interaction effect. That is, if worsening in PRO scores at Month 12 was due to non-sustainers, then changes in PRO scores should vary as a function of sustainer status, with remission non-sustainers showing worsening of PRO scores and with remission sustainers showing no change or improvements in PRO scores from Week 8 to Month 12.

#### Associations between changes in disease activity and PRO measures in the acute phase

The direction and magnitude of associations between changes in disease activity and PROs over the course of the acute phase were examined using Spearman rank-order correlation coefficients (ρ)^2^. Change scores were calculated for patients in the acute phase sample by subtracting baseline scores from final acute phase visit scores for all measures. Spearman coefficients were calculated between change scores for each PRO measure and change scores for the modified UC-DAI total.

CIs for each correlation coefficient were estimated using the following procedure. First, the correlation coefficient (ρ) was transformed into a *z*-score (z_ρ_) using Fisher’s *r*-to-*z* transformation [42]. Second, the standard error for *z*_ρ_ was calculated using the following equation from Fisher [43]:

$$ {SE}_{z_{\rho }} $$ *=* $$ \frac{1}{\sqrt{n-3.}} $$

Third, the 95% CI for z_ρ_ was calculated by multiplying ${SE}_{z_{\rho }}$ by 1.96, as indicated by Fisher [44]. Fourth, the 95% CI for z_ρ_ was transformed into the 95% CI for ρ using the inverse of Fisher’s *r*-to-*z* transformation [42].

Tests of whether the value of each correlation was statistically different from 0 were calculated using *P*-values across all measures.

#### Impact of missing data

At a number of trial sites, some or all PRO measures were improperly administered or not administered at all during the baseline visit; for this reason, baseline PRO scores from 94 acute phase sample patients (13.1%) were not usable for analysis. In addition, while translated versions of the SF-12v2 were available for all languages used by patients enrolled in this trial, translated versions for the language of some enrolled patients were not available for the SIBDQ (Kannada, Colombian Spanish) or the WPAI:UC (Indian English, Kannada, Punjabi, Colombian Spanish). This latter issue led to a virtual lack of any SIBDQ and WPAI:UC scores at any visit from patients who participated in any of the four trial sites in Colombia and patients at six of the 16 trial sites in India, and resulted in baseline SIBDQ scores being calculable for only 549 patients (76.6%) and baseline WPAI:UC scores calculable for only 322 patients (44.9%) in the acute phase sample. Because these data were missing due to systematic causes, and thus missing not at random (MNAR) [45], no methods for imputation of missing data at the visit level were applied in the primary analyses. However, the potential impact of missing PRO data on the results was examined using three sensitivity analyses. First, patient characteristics and PRO and UC-DAI scores of patients with missing SF-12v2, SIBDQ, and/or WPAI:UC scores for at least one visit during a phase, were compared to the corresponding data for patients who had non-missing scores on these scales at all visits. Specifically, six comparisons were made, by instrument (SF-12, IBDQ, and WPAI) and by sample (acute phase sample, maintenance phase sample). For the acute phase sample, for each instrument, baseline characteristics were compared between patients who had missing scores on all domains of the instrument during any acute phase visit and patients with non-missing values on at least one domain at all acute phase visit. Similarly, for the maintenance phase, baseline characteristics were compared between patients who had missing scores on all domains of the instrument during either maintenance phase visit and patients with non-missing values on at least one domain at both maintenance phase visits.

Second, a sensitivity analysis was conducted in which the MMRM analyses of each PRO measure were re-run for the subset of patients excluding all patients at the four Colombian sites and six Indian sites mentioned above.

Third, despite the fact that these data were MNAR, we applied multiple imputation that is generally used for missing at random (MAR) data to replace missing values of SIBDQ and SF-12v2 scores and the WPAI:UC Activity impairment domain (values for the other WPAI:UC domains were not imputed because most ‘missing’ data for these domains were due to the lack of patients’ employment). The multiple imputation included each UC-DAI component and WPAI:UC Absenteeism, Presenteeism, and Overall work impairment domains as predictor variables. SIBDQ and SF-12v2 scores and the WPAI:UC Activity impairment domain were simultaneously imputed and used as predictors. A total of five datasets using values from multiple imputation was used. Models from the primary analyses were then tested using data from the pooled dataset based on the average of values across all five datasets.

Data imputation methods used to substitute values at the item level due to missing responses for items on a measure followed developer recommendations for each instrument.

### Statistical software

Burden analyses were performed using SAS® version 9.2 for Windows (SAS Institute, Cary, NC, USA). Calculations of effect sizes were performed using Microsoft Excel® version 2007 (Microsoft, Redmond, WA, USA). Calculations of 95% CI around effect sizes were performed using the “BootES” package [46] in R. All other analyses were performed using SPSS® versions 17.02 and 23.0 for Windows (SPSS, Inc., Chicago, IL, USA).

## Results

### Baseline sample characteristics

Descriptive statistics for baseline sample characteristics, including demographics, clinical characteristics (modified UC-DAI component and total scores), and PRO scores for both the acute phase and maintenance phase samples, are presented in Table 1. Mean (SD) age of patients was approximately 43 (14) years, and a slight majority of patients were female (57%).

**Table 1:** Baseline characteristics for acute phase and maintenance phase samples

	Acute phase sample (*n* = 717)	Maintenance phase sample (*n* = 459)
Gender, *n* (%)		
Female	308 (43.0)	200 (43.6)
Male	409 (57.0)	259 (56.4)
Age, mean (SD)	42.9 (14.0)	42.7 (14.2)
BMI, mean (SD)	24.4 (4.9)	24.3 (4.8)
UC-DAI, mean (SD)		
Stool frequency (range: 0–3)	1.7 (0.8)	1.6 (0.8)
Rectal bleeding severity (range: 0–3)	1.3 (0.7)	1.2 (0.7)
Mucosal appearance (range: 0–3)	1.9 (0.5)	1.9 (0.5)
Physician global assessment (range: 0–3)	1.6 (0.5)	1.6 (0.5)
Total score (range: 0–12)	6.6 (1.6)	6.3 (1.5)
SF-12v2, mean (SD)		
Physical functioning	46.3 (9.1)	46.2 (9.1)
Role physical	43.8 (7.9)	44.5 (7.5)
Bodily pain	43.5 (9.1)	43.7 (8.7)
General health	41.5 (10.1)	41.1 (9.8)
Vitality	46.8 (9.5)	47.2 (8.9)
Social functioning	42.4 (9.3)	43.1 (8.9)
Role emotional	41.9 (9.6)	41.8 (9.4)
Mental health	44.2 (9.4)	44.4 (9.0)
PCS	44.9 (7.7)	45.0 (7.6)
MCS	43.1 (9.2)	43.5 (8.9)
SIBDQ, mean (SD)		
Bowel symptoms (range: 3–21)	12.6 (3.1)	12.8 (3.1)
Systemic symptoms (range: 2–14)	8.9 (2.6)	9.1 (2.5)
Emotional function (range: 3–21)	12.8 (3.9)	13.1 (3.8)
Social function (range: 2–14)	8.9 (3.0)	9.2 (2.9)
Total score (range: 10–70)	43.2 (10.6)	44.1 (10.4)
WPAI:UC, mean (SD); range (0–100)		
Absenteeism	13.3 (24.0)	12.3 (22.2)
Presenteeism	36.1 (24.5)	32.7 (22.8)
Overall work impairment	42.2 (28.2)	39.1 (26.6)
Activity impairment	41.5 (25.6)	38.9 (24.7)

*BMI* body mass index, *MCS* mental component summary, *PCS* physical component summary, *SD* standard deviation, *SF-12v2* SF-12v2 Health Survey, *SIBDQ* Short Inflammatory Bowel Disease Questionnaire, *UC-DAI* Ulcerative Colitis Disease Activity Index, *WPAI:UC* Work Productivity and Activity Impairment questionnaire for ulcerative colitis

### Burden analysis

**Fig. 2:**
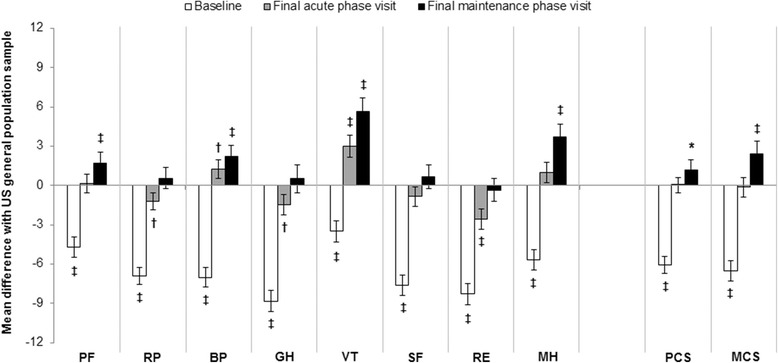
SF-12v2 score differences between trial patients and an age- and gender-matched US general population sample. PF: physical functioning; RP: role physical; BP: bodily pain; GH: general health; VT: vitality; SF: social functioning; RE: role emotional; MH: mental health; PCS: physical component summary; MCS: mental component summary. Error bars reflect 95% confidence intervals around mean differences. Significantly different from US general population sample based on *P*-values: * *P* < 0.05; † *P* < 0.01; ‡ *P* < 0.001

Differences between baseline mean SF-12v2 scores from acute phase sample patients with estimated mean scores of the age- and gender-matched US general population sample (Fig. 2, white bars) indicate a burden on all SF-12v2 scores, encompassing both physical and mental dimensions of HRQoL (all *P* < 0.001). Among domains, the smallest deficit observed was for VT (− 3.5 points), while GH (− 8.8 points) and RE (− 8.3 points) showed the greatest burden, with the burden for the remaining domains ranging from − 4.7 to − 7.7 points. The magnitude of burden was similar between PCS (− 6.1 points) and MCS (− 6.5 points).

At the final acute phase visit (Fig. 2. gray bars), acute phase sample patients’ scores were significantly below those of the US general population on RP, GH, and RE. The magnitude of differences for these three domains was small, with differences ≤ − 2.6 points. Scores on all other domains were either not statistically significantly different between the two samples or showed a statistically significant advantage for trial patients. Both PCS and MCS scores were virtually identical for trial patients and the US general population.

Maintenance phase sample patients’ SF-12v2 scores at the Month 12 visit were not statistically significantly different or significantly higher than those of the US general population on all domains (Fig. 2, black bars).

### Impact of treatment on PRO measures

Least squares (LS) mean SF-12v2 scores at baseline and final acute phase visits for patients in the acute phase sample with a score at both visits are presented in Table 2. LS mean scores for all domains and both PCS and MCS showed statistically significant increases from baseline to final acute phase visit (all *P* < 0.001). Effect sizes indicated that the magnitude of improvements were medium-sized for all domains, with the relatively smallest improvement observed for PF (*d*_*z*_ = 0.46) and the largest for BP (*d*_*z*_ = 0.81). Medium-sized effects for improvements in scores were observed for both PCS (*d*_*z*_ = 0.71) and MCS (*d*_*z*_ = 0.63).

**Table 2:** SF-12v2 and SIBDQ scores for the acute phase sample at acute phase visits

		Baseline		Week 8		Change	
	*N*	LS Mean	SE	LS Mean	SE	LS Mean Difference (95% CI)	Effect size *d*_*z*_, (95% CI)
SF-12v2							
Physical functioning	673	46.3	0.36	51.0	0.34	4.6‡ (3.7, 5.6)	0.41 (0.32, 0.49)
Role physical	673	43.8	0.32	49.4	0.30	5.6‡ (4.7, 6.5)	0.61 (0.52, 0.70)
Bodily pain	673	43.5	0.37	51.6	0.33	8.1‡ (7.2, 9.1)	0.74 (0.63, 0.83)
General health	674	41.6	0.40	48.8	0.37	7.3‡ (6.2, 8.3)	0.63 (0.54, 0.73)
Vitality	672	46.8	0.38	53.2	0.39	6.4‡ (5.3, 7.5)	0.53 (0.43, 0.61)
Social functioning	674	42.5	0.37	49.2	0.35	6.8‡ (5.8, 7.8)	0.63 (0.53, 0.71)
Role emotional	673	41.9	0.38	47.5	0.37	5.6‡ (4.6, 6.6)	0.49 (0.39, 0.58)
Mental health	674	44.2	0.38	50.8	0.39	6.6‡ (5.5, 7.6)	0.56 (0.47, 0.65)
PCS	673	44.9	0.31	50.8	0.28	5.9‡ (5.1, 6.8)	0.65 (0.55, 0.74)
MCS	674	43.2	0.37	49.5	0.36	6.4‡ (5.3, 7.4)	0.58 (0.49, 0.66)
SIBDQ							
Bowel symptoms	602	12.6	0.13	16.7	0.15	4.1‡ (3.7, 4.5)	1.00 (0.88, 1.10)
Systemic symptoms	602	8.9	0.11	10.9	0.11	2.0‡ (1.7, 2.3)	0.68 (0.57, 0.77)
Emotional function	602	12.8	0.17	16.1	0.17	3.2‡ (2.8, 3.7)	0.74 (0.64, 0.86)
Social function	601	9.0	0.13	11.6	0.12	2.6‡ (2.3, 3.0)	0.77 (0.66, 0.86)
Total score	600	43.3	0.45	55.3	0.49	12.0‡ (10.7, 13.3)	0.92 (0.80, 1.02)

*CI* confidence interval, *LS* least squares, *MCS* mental component summary, *PCS* physical component summary, *SE* standard error, *SF-12v2* SF-12v2 Health Survey, *SIBDQ* Short Inflammatory Bowel Disease Questionnaire

Findings for each outcome are based on mixed-effects models for repeated measures with subject as a random effect and visit as a fixed effect

Week 8 includes early withdrawal visits during the acute phase

‡*P* < 0.001

Mean SIBDQ domain and total scores at baseline and final acute phase visits for patients in the acute phase sample with a score at both visits are also presented in Table 2. Means for all domains and total score were significantly larger at final acute phase visit than at baseline (all *P* < 0.001). Effect sizes show that the magnitude of improvements in domain scores ranged from medium-sized (0.68 for Systemic symptoms) to large (1.00 for Bowel symptoms), with a large effect observed for the SIBDQ total score (*d*_*z*_ = 0.92).

Mean WPAI:UC domain scores at baseline, Week 3, and final acute phase visits for patients in the acute phase sample are presented in Table 3. Statistically significant differences in means across visits were observed for all domains (all *P* < 0.001). Post hoc tests for pairwise differences in mean scores found statistically significant decreases (i.e., improvements) in all domains from baseline to both Week 3 and final acute phase visits (all Bonferroni-adjusted *P* < 0.001). Pairwise comparisons of mean scores also found significant decreases from Week 3 to final acute phase visit for Presenteeism, Overall work impairment, and Activity impairment domains (Bonferroni-adjusted *P* < 0.001 for all differences), while scores on the Absenteeism domain did not statistically differ between these two visits.

**Table 3:** WPAI:UC scores for the acute phase sample at acute phase visits

		Baseline		Week 3		Week 8			Post hoc comparisons (Bonferroni-adjusted *P* [α/3])		
	*N*	LS Mean	SE	LS Mean	SE	LS Mean	SD	*P*	Baseline vs. Week 3	Baseline vs. Week 8	Week 3 vs. Week 8
Absenteeism	367	13.0	1.35	6.6	1.03	4.5	0.80	‡	‡	‡	0.1341
Presenteeism	376	35.9	1.37	21.2	1.15	16.7	1.19	‡	‡	‡	‡
Overall work impairment	365	42.0	1.60	25.4	1.42	19.8	1.39	‡	‡	‡	‡
Activity impairment	579	41.5	1.12	26.2	1.02	19.3	1.02	‡	‡	‡	‡

*LS* least squares, *SE* standard error, *WPAI:UC* Work Productivity and Activity Impairment questionnaire for ulcerative colitis

Findings for each outcome are based on mixed-effects models for repeated measures with subject as a random effect and visit as a fixed effect

Week 8 includes early withdrawal visits during the acute phase

‡*P* < 0.001

Mean SF-12v2 scores at baseline, Week 8, and Month 12 visits for patients in the maintenance phase sample are presented in Table 4. Statistically significant differences in mean scores across visits were observed for all domains and both PCS and MCS (all *P* < 0.001). Post hoc tests for pairwise differences in mean scores found statistically significant increases in all scores from baseline to both Week 8 and Month 12 visits (all Bonferroni-adjusted *P* < 0.001). Comparisons of scores between Week 8 and Month 12 visits found no significant mean differences for seven of the eight domains, nor for PCS and MCS; significant decreases in mean scores were observed for BP (Bonferroni-adjusted *P* < 0.01).

**Table 4:** SF-12v2 and SIBDQ scores for the maintenance phase sample at acute and maintenance phase visits

		Baseline		Week 8		Month 12			Post hoc comparisons (Bonferroni-adjusted *P* [α/3])		
	*N*	LS Mean	SE	LS Mean	SE	LS Mean	SE	*P*	Baseline vs. Week 8	Baseline vs. Month 12	Week 8 vs. Month 12
SF-12v2											
Physical functioning	456	46.3	0.46	52.6	0.36	52.6	0.40	‡	‡	‡	1.0000
Role physical	456	44.5	0.38	51.7	0.29	51.2	0.39	‡	‡	‡	0.8573
Bodily pain	455	43.7	0.44	54.1	0.27	52.6	0.41	‡	‡	‡	†
General health	456	41.2	0.49	51.1	0.38	50.9	0.53	‡	‡	‡	1.0000
Vitality	456	47.3	0.45	55.7	0.42	56.0	0.51	‡	‡	‡	1.0000
Social functioning	456	43.1	0.45	51.8	0.34	50.7	0.44	‡	‡	‡	0.0666
Role emotional	456	41.9	0.47	49.9	0.35	49.8	0.42	‡	‡	‡	1.0000
Mental health	456	44.5	0.45	53.5	0.39	53.7	0.48	‡	‡	‡	1.0000
PCS	456	45.0	0.38	52.6	0.27	52.0	0.37	‡	‡	‡	0.3256
MCS	456	43.6	0.45	52.2	0.36	52.3	0.44	‡	‡	‡	1.0000
SIBDQ											
Bowel symptoms	406	12.8	0.17	18.1	0.13	17.3	0.20	‡	‡	‡	‡
Systemic symptoms	405	9.1	0.14	11.5	0.11	11.4	0.13	‡	‡	‡	0.6393
Emotional function	404	13.1	0.21	17.4	0.16	17.0	0.21	‡	‡	‡	0.1070
Social function	406	9.2	0.16	12.6	0.10	12.2	0.14	‡	‡	‡	*
Total score	404	44.2	0.57	59.7	0.43	57.8	0.63	‡	‡	‡	†

*LS* least squares, *MCS* mental component summary, *PCS* physical component summary, *SE* standard error, *SF-12v2* SF-12v2 Health Survey, *SIBDQ* Short Inflammatory Bowel Disease Questionnaire

Findings for each outcome are based on mixed-effects models for repeated measures with subject as a random effect and visit as a fixed effect

Week 8 includes early withdrawal visits during the acute phase. “Month 12” includes early withdrawal visits during the maintenance phase

*P < 0.05, †*P* < 0.01, ‡*P* < 0.001

Mean SIBDQ domain and total scores at baseline, Week 8, and Month 12 visits for patients in the maintenance phase sample are also presented in Table 4. Statistically significant differences in means were observed across visits for all domains and total scores (all *P* < 0.001). Post hoc tests for pairwise differences found statistically significant improvement in all mean scores from baseline to both Week 8 and Month 12 visits (all Bonferroni-adjusted *P* < 0.001). Comparisons of mean scores between Week 8 and Month 12 visits found significant decreases for Bowel symptoms (Bonferroni-adjusted *P* < 0.001) and Social function domains (Bonferroni-adjusted *P* < 0.05), but no significant differences were observed for the Systemic symptoms or Emotional function domains. A significant decrease from Week 8 to Month 12 visit was observed for total score (Bonferroni-adjusted *P* < 0.01).

Mean WPAI:UC domain scores at baseline, Week 3, Week 8, and Month 12 visits for patients in the maintenance phase sample are presented in Table 5. Statistically significant differences in means across visits were observed for all domains (all *P* < 0.001). Post hoc tests for pairwise differences in mean scores found statistically significant decreases (i.e., improvements) in all domains from baseline to Week 3, Week 8, and Month 12 visits (all Bonferroni-adjusted *P* < 0.001). Comparisons of scores between Week 8 and Month 12 visits found significant increases (i.e., worsening) in mean scores for Activity impairment (Bonferroni-adjusted *P* < 0.01), but no statistically significant changes in mean scores for Presenteeism, Absenteeism, or Overall work impairment.

**Table 5:** WPAI:UC scores for the maintenance phase sample at acute and maintenance phase visits

		Baseline		Week 3		Week 8		Month 12			Post hoc comparisons (Bonferroni-adjusted *P* [α/4])			
	*N*	LS Mean	SE	LS Mean	SE	LS Mean	SE	LS Mean	SE	*P*	Baseline vs. Week 3	Baseline vs. Week 8	Baseline vs. Month 12	Week 8 vs. Month 12
Absenteeism	260	12.1	1.60	5.2	1.19	2.6	0.78	3.8	0.93	‡	‡	‡	‡	1.0000
Presenteeism	264	32.6	1.62	16.3	1.17	9.6	0.96	13.4	1.44	‡	‡	‡	‡	0.1015
Overall work impairment	260	38.9	1.92	20.1	1.54	11.5	1.24	15.7	1.72	‡	‡	‡	‡	0.2222
Activity impairment	395	38.9	1.36	20.6	1.05	10.7	0.78	15.4	1.17	‡	‡	‡	‡	†

*LS* least squares, *SE* standard error, *WPAI:UC* Work Productivity and Activity Impairment questionnaire for ulcerative colitis

Findings for each outcome are based on mixed-effects models for repeated measures with subject as a random effect and visit as a fixed effect

Week 8 includes early withdrawal visits during the acute phase. “Month 12” includes early withdrawal visits during the maintenance phase

†*P* < 0.01, ‡*P* < 0.001

### Remission sustainer analysis

Changes in LS Mean SF-12v2, SIBDQ, and WPAI:UC domain scores from Week 8 to Month 12 for patients in the maintenance phase sample who sustained or failed to sustain remission at Month 12 are presented in Table 6. For each domain and summary score of each instrument, a statistically significant interaction effect for sustainer status by visit was observed, indicating that changes in each score across visit differed as a function of sustainer status. Comparisons within each group found that patients who did not sustain remission exhibited statistically significant worsening in all scores from Week 8 to Month 12 (all Bonferonni-adjusted *P* < 0.001), while patients who sustained remission showed either no change or further improvements in scores.

**Table 6:** Change in PRO scores from Week 8 to Month 12 for patients in the maintenance phase sample who sustained or did not sustain remission at Month 12

	Did not sustain remission		Sustained remission		
	N	Change in LS Mean (SE)	N	Change in LS Mean (SE)	*P* ^*a*^
SF-12v2, mean (SD)					
Physical functioning	110	−4.2 (0.79)‡	225	1.6 (0.56)*	‡
Role physical	109	−6.0 (0.73)‡	225	1.6 (0.51)*	‡
Bodily pain	110	−7.2 (0.76)‡	223	0.8 (0.53)	‡
General health	110	−8.9 (0.88)‡	225	3.0 (0.62)*	‡
Vitality	110	−5.2 (1.00)‡	221	2.5 (0.71)*	‡
Social functioning	110	−6.3 (0.82)‡	225	1.0 (0.58)	‡
Role emotional	110	−4.9 (0.78)‡	223	1.8 (0.55)*	‡
Mental health	110	−5.1 (0.87)‡	225	2.0 (0.62)*	‡
PCS	110	−6.3 (0.67)‡	225	1.5 (0.48)*	‡
MCS	110	−4.9 (0.77)‡	225	1.9 (0.54)*	‡
SIBDQ, mean (SD)					
Bowel symptoms	95	−4.1 (0.33)‡	204	0.4 (0.23)	‡
Systemic symptoms	96	−1.9 (0.24)‡	206	0.5 (0.17)*	‡
Emotional function	95	−3.2 (0.32)‡	205	0.6 (0.22)*	‡
Social function	96	−2.7 (0.24)‡	208	0.5 (0.16)*	‡
Total score	92	−12.0 (0.96)‡	199	1.8 (0.67)*	‡
WPAI:UC, mean (SD)					
Absenteeism	54	9.3 (2.17)‡	113	−1.6 (1.54)	‡
Presenteeism	58	19.6 (2.42)‡	117	−2.5 (1.75)	‡
Overall work impairment	54	25.4 (3.00)‡	111	−3.7 (2.13)	‡
Activity impairment	92	22.3 (2.04)‡	199	−2.4 (1.42)	‡

*LS* least squares, *MCS* mental component summary, *PCS* physical component summary, *PRO* patient-reported outcome, *SD* standard deviation, *SE* standard error, *SF-12v2* SF-12v2 Health Survey, *SIBDQ* Short Inflammatory Bowel Disease Questionnaire, *WPAI:UC* Work Productivity and Activity Impairment questionnaire for ulcerative colitis

Findings for each outcome are based on mixed-effects models for repeated measures with subject as a random effect, and sustainer status, visit, and the sustainer status x visit interaction as fixed effects

Week 8 includes early withdrawal visits during the acute phase. “Month 12” includes early withdrawal visits during the maintenance phase

^a^Indicates statistical significance of the sustainer status x visit interaction effect

*P < 0.05, ‡*P* < 0.001

### Association between changes in disease activity and PRO measures in the acute phase

Table 7 presents Spearman correlation coefficients (ρ) with 95% CIs for change from baseline to Week 8 visit scores from each PRO measure and the modified UC-DAI total score for patients in the acute phase sample. All correlations were statistically different from 0 (all *P* < 0.001) in the predicted directions, with decreases (improvements) in UC-DAI scores inversely associated with increases in SF-12v2 and SIBDQ scores, and positively correlated with decreases in WPAI:UC scores. The magnitude of correlations with UC-DAI scores ranged from small to moderate for each PRO instrument, with coefficients ranging from − 0.31 (MH) to − 0.44 (RP) for SF-12v2 domains (median ρ = − 0.40), from − 0.33 (Systemic symptoms) to − 0.52 (both Bowel symptoms and Emotional function) for SIBDQ domains (median ρ = − 0.50), and from 0.31 (Absenteeism) to 0.48 (Activity impairment) for WPAI:UC domains (median ρ = 0.40).

**Table 7:** Spearman correlation coefficients between changes in PRO scores and in UC-DAI total scores from baseline to Week 8 in the acute phase

	N	ρ (95% CI)
SF-12v2		
Physical functioning	532	−0.32 (−0.39, −0.24)
Role physical	527	−0.44 (− 0.51, − 0.37)
Bodily pain	522	− 0.41 (− 0.48, − 0.34)
General health	532	− 0.40 (− 0.47, − 0.33)
Vitality	523	− 0.35 (− 0.42, − 0.27)
Social functioning	532	− 0.43 (− 0.50, − 0.36)
Role emotional	529	− 0.40 (− 0.47, − 0.33)
Mental health	532	−0.31 (− 0.38, − 0.23)
PCS	531	− 0.43 (− 0.50, − 0.36)
MCS	531	−0.37 (− 0.44, − 0.29)
SIBDQ		
Bowel symptoms	442	−0.52 (− 0.58, − 0.45)
Systemic symptoms	458	−0.33 (− 0.41, − 0.25)
Emotional function	453	−0.47 (− 0.54, − 0.39)
Social function	461	−0.52 (− 0.58, − 0.45)
Total score	430	−0.53 (− 0.59, − 0.46)
WPAI:UC		
Absenteeism	223	0.31 (0.19, 0.42)
Presenteeism	229	0.36 (0.24, 0.47)
Overall work impairment	218	0.44 (0.33, 0.54)
Activity impairment	418	0.48 (0.40, 0.55)

*CI* confidence interval, *MCS* mental component summary, *PCS* physical component summary, *PRO* patient-reported outcomes, *SF-12v2* SF-12v2 Health Survey, *SIBDQ* Short Inflammatory Bowel Disease Questionnaire, *UC-DAI* Ulcerative Colitis Disease Activity Index, *WPAI:UC* Work Productivity and Activity Impairment questionnaire for ulcerative colitis

Week 8 includes early withdrawal visits during the acute phase

All correlation coefficients are statistically different from 0 (all *P* < 0.001)

### Impact of missing data

Descriptive analyses (data not shown) found no evidence for meaningful or consistent differences in age, gender, UC activity (i.e., UC-DAI scores), or PRO scores between patients who were missing data on at least one PRO during at least one visit within a phase and patients with PRO data at all visits of a phase. Noticeable and consistent differences between subgroups were observed with respect to patients’ race, ethnicity, and country, with the patterns consistent with the fact that the majority of missing PRO scores were from the identified Colombian and Indian trial sites.

Sensitivity analyses in which we tested MMRM models for the effect of treatment on PROs over time (i.e., results presented in Tables 2, 3, 4 and 5) when excluding all patients from the problematic Colombian and Indian sites (129 patients in the acute phase efficacy population and 91 patients in the maintenance phase efficacy population were excluded) produced findings (data not shown) much like those observed in the primary analysis. Across all tests, a qualitatively different result (i.e., an effect that was statistically significant in the primary analysis but non-significant in the sensitivity analysis, or vice versa) was not observed for any test of the visit effect in the models. The only qualitative differences observed were the following: changes from Week 8 to Month 12 for the Social Functioning domain and PCS of the SF-12v2 and the Emotional Function domain of the SIBDQ were statistically significant in the sensitivity analysis (all Bonferroni-adjusted *P* < 0.05) but not in the primary analysis (Bonferroni-adjusted *P* > 0.05). Results from the sensitivity analysis therefore indicated that the exclusion of patients from sites where PRO measures were not administered per protocol had a minimal influence on our findings.

Sensitivity analyses in which we tested MMRM models for the effect of treatment on PROs over time (i.e., results presented in Tables 2, 3, 4 and 5) when using datasets produced by multiple imputation also yielded findings (data not shown) much like those observed in the primary analysis. Across all tests, a qualitatively different result was not observed for any test of the visit effect in the models. The only qualitative differences observed were the following: changes from Week 3 to Week 8 for the WPAI:UC Activity impairment domain was statistically significant in the primary analysis (Bonferroni-adjusted *P* < 0.001) but not in the sensitivity analysis (Bonferroni-adjusted *P* = 0.194); and changes from Week 8 to Month 12 for the Social Function domain and Total score of the SIBDQ and the Activity Impairment domain of the WPAI:UC were statistically significant in the primary analysis (all Bonferroni-adjusted *P* < 0.05) but not in the sensitivity analysis (Bonferroni-adjusted *P* > 0.05).

## Discussion

Previous studies have established that patients with UC show deficits in both physical and mental aspects of HRQoL, that the presence and magnitude of these deficits are associated with disease activity (e.g., symptom severity, remission status), that treatment of active UC is associated with improvements in HRQoL, and that continued long-term treatment is associated with sustained improvements in HRQoL. Findings from the current analysis are mostly consistent with each of these established relations. Baseline SF-12v2 scores from the trial sample were significantly lower (worse) than scores from a matched US general population sample across all domains, indicating baseline burden of disease. Following eight weeks of daily treatment with 4.8 g/day of multimatrix mesalamine, patients with UC showed significant improvement in all measured domains of generic and disease-specific HRQoL. These improvements in HRQoL were associated with decreases in disease activity, as indicated by significant, small-to-moderate negative correlations between all SF-12v2 and SIBDQ domains and UC-DAI scores. These improvements were also of sufficient magnitude to eliminate the burden of UC on most measured aspects of HRQoL: at Week 8 visit, SF-12v2 scores from the trial sample were not statistically significantly different from (or were statistically better than) scores from the general population sample on five of eight domains (all but RP, GH, and RE) and both PCS and MCS. Patients with UC who subsequently received 12 months of daily treatment with 2.4 g/day of multimatrix mesalamine maintained these improvements on some, though not all, domains: scores at the Month 12 visit showed no significant decreases from Week 8 scores for six of eight SF-12v2 domains (all but BP and SF) and both PCS and MCS, and for the Systemic symptoms domain of the SIBDQ. By the end of maintenance treatment, the burden of UC was eliminated for all measured aspects of HRQoL, as SF-12v2 scores from the trial sample were not statistically significantly different from, or were statistically significantly better than, scores from the general population sample on all domains and both PCS and MCS.

Prior research has also established disease activity as a predictor of work-related outcomes for patients with UC [10, 11, 16, 27–29], which was supported by our findings of significant, small-to-moderate correlations between all WPAI:UC domains and UC-DAI scores. However, no studies in the published literature present data that assess the impact of treatment on work-related outcomes for patients with UC. This analysis provides the first results showing improvements in the impact of patients’ UC on multiple aspects of work-related outcomes, including decreased absenteeism and increased work productivity, following treatment. Further, improvements on two of the four WPAI:UC domains, Absenteeism and Overall work impairment, were sustained during the 12-month maintenance phase.

While improvements in many HRQoL and work-related outcome domains were sustained during the maintenance phase, statistically significant worsening during this phase was observed for others, including two of the four SIBDQ domains and SIBDQ total score. The sustainer analysis found that for patients still in complete or partial remission at Month 12, scores on PRO measures were either unchanged or showed even further improvements, while scores for patients who were no longer in remission at Month 12 significantly worsened for all outcomes. These results are consistent with previous evidence showing the association between remission status and HRQoL [47–49] and work-related outcomes [16, 28] in patients with UC. The current findings that decreased severity of symptoms in the acute phase predicted improved HRQoL and less impairment in work-related outcomes lends further support for the relation between disease activity and PROs.

The burden of UC on HRQoL and work-related outcomes observed in this analysis reflects documented experiences of patients with IBD. Interviews and focus groups with patients have found serious concerns regarding lack of control over their bodily functions, disease progression, and the possibility of not having immediate access to a toilet [50–52]. These problems lead to impairments in their work productivity [50, 53, 54] and limit their ability to engage in social and recreational activities [50, 51, 54, 55]. Impairments in work and other activities subsequently hinder their achievement and ability to develop and maintain relationships with other people, resulting in feelings of isolation and depression [53, 54]. Given this impact of disease symptoms on patients’ experiences, the improvements of patients’ HRQoL and WPAI scores that accompany the decrease in disease activity following eight weeks of daily treatment with 4.8 g/day of multimatrix mesalamine in the current study should come as little surprise.

Poor work-related outcomes, including lost productivity, unemployment, and work disability, that accompany active UC result in significant indirect costs; estimates of these costs in the US have ranged from approximately $2000–$9000 (US 2008) per patient per year [25, 56–58]. Because the current analysis showed increased work productivity and decreased absenteeism for patients with UC who were treated with multimatrix mesalamine, use of this treatment could potentially reduce some of these indirect costs. While several studies have used cost-effectiveness modelling techniques to assess direct costs of mesalamine treatment for patients with UC [59–65], their models have not included the impact of work-related outcome improvements on indirect costs. Future studies are thus needed to determine the overall economic benefits in work-related outcomes that are associated with this treatment regimen.

There are several limitations of this analysis that should be taken into account when interpreting its findings. The use of open-label design, rather than a randomized, controlled, blinded design, increased the ecological validity of the trial by more closely matching the manner in which treatment is administered and used in real-world settings. However, this design decreases the ability to draw causal inferences about the impact of treatment on HRQoL and work-related outcomes, and the mechanistic role of disease activity, on these outcomes. While we observed improvements in HRQoL and work-related outcomes following treatment with multimatrix mesalamine, as well as associations between these improvements and changes in disease activity, it cannot be concluded that the treatment produced these improvements, or whether changes in disease activity moderated the effect of treatment on these outcomes. The lack of comparison with a placebo control group in this study also limits inferences regarding the efficacy of multimatrix mesalamine, particularly given that findings from placebo-controlled studies of oral mesalamine treatment for UC show a non-zero rate of induction and maintenance of remission in patients receiving placebo treatment [66, 67]. Future trials incorporating randomized, controlled, blinded designs would provide a more robust understanding of the effectiveness of multimatrix mesalamine on improving HRQoL and work-related outcomes for patients with UC, and the role of disease activity within these relationships.

The number of missing PRO scores at the baseline visit due to administrative causes might also limit the interpretability of these findings. Because the patient characteristics upon which missingness was based (e.g., country, language) might have been independently related to key demographic characteristics, disease activity, HRQoL, and/or work-related outcomes, patients with and without missing data by instrument and sample were compared, and sensitivity analyses were conducted to see if using multiple imputation techniques to generate values for missing scores and excluding patients who participated at the 10 sites where these administrative issues occurred impacted our findings. Based on the trivial differences on these outcomes between subgroups of patients and between results of primary and sensitivity analyses, we believe that missing data most likely did not have a substantial impact on our results, although this cannot be dismissed with absolute certainty.

Another limitation of this analysis was the use of US general population norms as the only benchmark sample for examining the burden of UC on HRQoL. Only 13% of patients in the acute phase sample and only 8% of patients in the maintenance phase sample were from the US, which means that scores for the vast majority of patients in these samples were compared to norms from a country other than their own. Examination of country-specific differences in scores on the SF-36 health survey, from which the SF-12 was adapted, showed substantial agreement in scores across 10 North American and Western European countries [68], indicating that US norms are appropriate for assessing burden for some non-US responders. However, the current trial included patients from countries outside of North America and Western Europe, including India, which was most heavily represented among countries, accounting for 27% and 30% of the acute phase and maintenance phase samples, as well as several Eastern European, African, and South American countries. The degree to which the general population normative scores for patients from these countries align with US general population norms has not been established, and the potential deviations in norms between countries could shift the values for what would be considered average scores. These country-based distinctions in norms may account for the fact that trial patients at Month 12 visit exhibited above-average scores on several SF-12v2 domains and both PCS and MCS. Results from the burden analysis presented here should be interpreted cautiously in light of the lack of country-specific norms used for comparison of trial patients’ SF-12v2 scores, and future research should address this issue.

## Conclusions

Patients with moderate-to-severe UC who received eight weeks of daily treatment with 4.8 g/day of multimatrix mesalamine showed improvements in all measured aspects of both HRQoL and work-related outcomes. Further, patients in partial or complete clinical and endoscopic remission who subsequently received 12 months of daily treatment with 2.4 g/day of multimatrix mesalamine maintained most of these improvements over this time frame. Improvements in HRQoL and work-related outcomes during the initial treatment phase and any reverses in these improvements during the maintenance phase were linked to concurrent changes in disease activity.

## Abbreviations

ANOVA: Analysis of Variance
BMI: Body Mass Index
BP: Bodily Pain
GH: General Health
HRQoL: Health-related Quality of Life
IBD: Inflammatory Bowel Disease
MCS: Mental Component Summary
MH: Mental Health
MMRM: Mixed-effects Models for Repeated Measures
MNAR: Missing Not At Random
PCS: Physical Component Summary
PF: Physical Functioning
PRO: Patient-reported Outcomes
QD: Once Daily
RE: Role limitations due to Emotional health problems
RP: Role limitations due to Physical health problems
SD: Standard Deviation
SF: Social Functioning
SF-12v2: SF-12v2® Health Survey
SIBDQ: Short Inflammatory Bowel Disease Questionnaire
UC: Ulcerative Colitis
UC-DAI: Ulcerative Colitis Disease Activity Index
US: United States
VT: Vitality
WPAI:UC: Work Productivity and Activity Impairment, Ulcerative Colitis-specific Version

## Footnotes

1 Whereas the original version of the UC-DAI [31] treated evidence for mucosal friability as an indicator of mild disease (i.e., endoscopy rating of 1), the modified version [69] treats it as an indicator of moderate disease (i.e., endoscopy rating of 2).

2 Non-parametric Spearman coefficients, rather than Pearson coefficients, were used to estimate associations because scores on some of the outcomes measures were not normally distributed.
